# Dynamics analysis of neural univariate time series by recurrence plots

**DOI:** 10.1186/1471-2202-16-S1-P105

**Published:** 2015-12-18

**Authors:** Tamara Tošić, Peter beim Graben, Kristin K Sellers, Flavio Fröhlich, Axel Hutt

**Affiliations:** 1 Inria, CNRS, Loria, UMR nº 7503, Vandœuvre-lès-Nancy, F-54500, France; 2 Institute of German Studies and Linguistics, Humboldt-University, 10178, Berlin, Germany; 3 Bernstein Center for Computational Neuroscience, 10178, Berlin, Germany; 4 University of North Carolina at Chapel Hill, Chapel Hill, 27599, NC, USA

## 

Transients in non-linear biological signals (e.g., population dynamics or physiological signals) encode an intrinsic behaviour of system dynamics. We study the problem of detecting dynamical transients given a set of signal trials. In general case, different biological signals emerge from different origins and hence exhibit distinct properties that are hard to grasp. For example, to analyze sleep recordings, one considers rhythms of the brain, the cardio-vascular and the respiratory systems. The synchronous analysis of the corresponding time series is an unsolved problem and extracting information from such signals and their trial statistics is challenging. In addition, measurement noise and time jitters between trials may corrupt signals. To attack this demanding problem, we start by a preliminary study of extracting features in multiple trials from univariate time series of the same origin, but without taking into account the common origin. The new method jointly analyzes neural signals by extracting statistical properties, obtained by exploiting the fundamental feature of dynamical systems, the recurrence structure.

**Figure 1:**
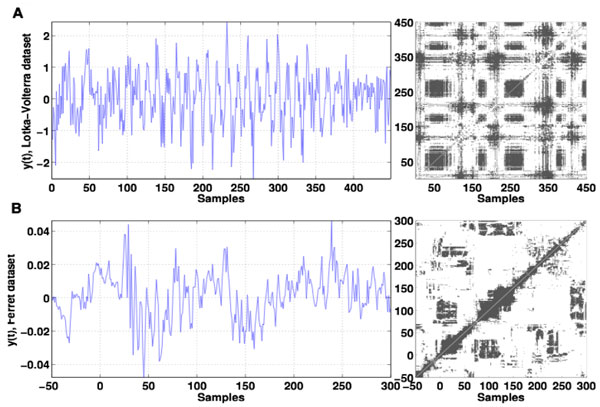
**Detection of dynamical transients**. (A) Superposition of several transient oscillations of different frequencies. (B) experimental Local Field Potentials measured in ferret visual cortex in the presence of a periodic 1Hz visual stimulus [3] starting at sample 0 with a period of 100 samples. Time series are given in left column. Statistically significant data (white values) of recurrence points are illustrated in right column figures (Chi-square test, p = 0.1).

Recurrences represent instances in time when the system's trajectory returns to a phase space neighborhood of a location it has visited previously, cf. [1]. Classical recurrence plots (RP) [2] represent symmetric binary valued matrices and characterise the system's phase space. Since neural recurrence patterns occur in particular frequency bands, we propose to build novel, frequency-selective recurrence plots from their time-frequency signal representations. To evaluate the recurrence results, we compute surrogate RPs by time randomisation of the original time-frequency representations. To obtain statistically significant recurrence information, statistics of the original RPs is pixel-wise compared with the surrogate dataset statistics. This novel inference test provides statistically significant pixel values and the corresponding recurrence structure. Results in Figure 1 show an artificial and neural time series and corresponding statistically significant recurrence plots. One observes that the method extracts well several transient oscillations of different frequencies. The analysis of the ferrets data reveals that the first stimulation response at t = 0ms is recurrent to the second response one stimulation period later, while the third response at t = 3s is different from the first two. This result indicates temporal neural adaptation during the stimulation.
